# RETRACTION NOTICE: REDUCING MOTION ARTIFACTS IN 4D MR IMAGES USING PRINCIPAL COMPONENT ANALYSIS (PCA) COMBINED WITH LINEAR POLYNOMIAL FITTING MODEL

**DOI:** 10.1120/jacmp.v16i3.5738

**Published:** 2015-05-08

The *Journal of Applied Clinical Medical Physics* announces that the article “Reducing motion artifacts in 4D MR images using principal component analysis (PCA) combined with linear polynomial fitting model,” by Juan Yang, Hongjun Wang, Yong Yin, and Dengwang Li has been retracted.

The *JACMP* has issued the following retraction statement:

The *Journal of Applied Clinical Medical Physics* retracts the article “Reducing motion artifacts in 4D MR images using principal component analysis (PCA) combined with linear polynomial fitting model,” which was published in this Journal in the March/April issue, Volume 16, Number 2 (2015). This retraction comes at the request of the authors, following an admission that the scientific data was improperly obtained and reported. The request signed by the authors can be viewed here:

http://www.jacmp.org/index.php/jacmp/article/view/5165/pdf_300

The Editor‐in‐Chief reviewed all aspects of the submission, and the peer‐review process, and found the review by the *JACMP* was proper in every respect. There was no indication within the manuscript that the data had its origin in an institution other than that of the four authors.

The Editor‐in‐Chief wishes to acknowledge the cooperation of the corresponding author in this matter, and commends him for his commitment to the scientific process.

## Supporting information

Supplementary Material

