# Supplementary material for: RETRACTION NOTICE: REDUCING MOTION ARTIFACTS IN 4D MR IMAGES USING PRINCIPAL COMPONENT ANALYSIS (PCA) COMBINED WITH LINEAR POLYNOMIAL FITTING MODEL
Source: J Appl Clin Med Phys. 2015 May 8;16(3):8. doi: 10.1120/jacmp.v16i3.5738 (PMC5690117; doi:10.1120/jacmp.v16i3.5738)
Supplement: Supplementary file 1 — Supplementary Material [file ACM2-16-008-s001.pdf]

Retraction Notification

We (authors), Juan Yang Hongjun Wang Yong Yin and Dengwang Li, are sorry to inform you that we want to retract the accepted paper ID (# 5165) titled 'Reducing motion artifacts in 4D MR images using principal component analysis (PCA) combined with linear polynomial fitting model', which has been accepted by *Journal of Applied Clinical Medical Physics*.

The first author (Juan Yang) was a visiting student in Duke University during doing this project and the patient data used in this paper came from Duke, however, the related person in Duke were not pleased with this after they found the published paper online.

After the authors carefully discussed, we determined to give up this good opportunity if possible. We appreciated great efforts the editorial team and the reviewers have made for the publication of this article.

We will still submit our contribution to your journal near future.

SIGNATURE: Juan Yang Hongjun Wang Yong Yin Dengwang Li

DATE: 3/22/2015

If you have any problem, please contact:

E-mail: hjw@sdu.edu.cn juanyang1002@126.com
